# Supplementary material for: Reverse engineering approach for improving the quality of mobile applications
Source: PeerJ Comput Sci. 2019 Aug 19;5:e212. doi: 10.7717/peerj-cs.212 (PMC7924421; doi:10.7717/peerj-cs.212)
Supplement: Supplemental Information 1 — The numbers from 1 to 29 present the mobile apps. From 1 to 5 in the second column present the anti-patterns group in each app with order (Attributes anti-patterns, Namespace anti-Patterns, Operation anti-patterns, Association anti-patterns, and Class anti-patterns). This table is for doing the one-way ANOVA test using SPSS. [file peerj-cs-05-212-s001.docx]

| Mobile App Name | Size(MB) | Downloads |
| --- | --- | --- |
| Test DPC 4.0.5 | 3.14 MB | 1.076.791 |
| Avast 6.5.3 Security | 20.71 MB | 1.364 |
| Free-Calls-Messages | 31.59 MB | 1.537 |
| Beautiful Gallery 2.3 | 11.31 MB | 497 |
| Play Store 9.3.4 | 14.17 MB | 6.950 |
| Wall Paper 1.2.166 | 2.29 MB | 9.730 |
| Oasis-Feng/Island 2.5 | 2.34 MB | 822 |
| Netflix-5-4-0-Build | 18.81 MB | 22.043 |
| Remainder 1.4.02 | 9.36 MB | 3.612 |
| Sound-Picker 8.0.0 | 3.9 MB | 2.142 |
| Air-Command 2.5.15 | 0.82 MB | 1.747 |
| Lifesum-Healthy-Lifestyle | 31.4 MB | 3.594 |
| Background-Defocus 2.2.9 | 3.45 MB | 2.960 |
| Gasbuddy-Find-Cheap-Gas | 29.64 MB | 334 |
| Soundcloud -Music-Audio.03.03 | 33.2 MB | 2,066 |
| Network-Monitor-Mini 1.0.197 | 2.88 MB | 307 |
| Casper Android 1.5.6.6 | 18.77 MB | 383.765 |
| Line 8.4.0 | 70.25 MB | 260 |
| Diagnosises | 6.96 MB | 36 |
| Viber 7.7.0.21 | 38.4 MB | 1.628 |
| Whats App 2.17.235 | 35.81 MB | 28.978 |
| Firefox 56.0 | 40.62 MB | 20.423 |
| Blue- Email And Calendar 1.9.3.21 | 43.2.4 MB | 203 |
| Google Camera 5.1.011.17 | 36.48 MB | 211.822 |
| You Tube 13.07 | 24.13 MB | 23.667 |
| True Caller 8.84.12 | 23.09 MB | 609 |
| Samsung Gallery 5.4.01 | 17.61 MB | 10.712 |
| Twitter 7.48.0 | 35.82 MB | 694 |
| Chrome Browser 66.0.3359 | 41.51 MB | 29.129 |

The features of the mobile apps which were downloaded from APK Mirror

For the evaluation of the proposed method

|  | **Mobile App** | **CHSO** | **NHSN** | **NLAD** | **NLAA** | **GBUE** | **CHSA** | **MMITMM** | **PACPP** | **SAAC** | **TDHPS** | **BinOver** | **AC** | **RS** | **RelComp** | **ImpAbs** | **Total** |
| --- | --- | --- | --- | --- | --- | --- | --- | --- | --- | --- | --- | --- | --- | --- | --- | --- | --- |
| 1 | Test  DPC 4.0.5 | 7 | 2 | 1 |  |  | 2 |  | 1 |  |  | 10 | 6 | 1 |  |  | **30** |
| 2 | Avast Android Mobile Security | 149 | 15 |  | 2 |  | 58 | 3 |  | 2 |  | 6 |  |  | 2 | 3 | **240** |
| 3 | Free-Calls-Messages | 4 | 1 | 2 |  | 2 | 1 | 1 |  |  |  | 3 | 2 |  |  |  | **16** |
| 4 | Beautiful Gallery 2.1 | 5 | 2 |  |  |  |  |  | 1 |  |  |  |  |  |  |  | **8** |
| 5 | Play  Store | 8 | 1 | 1 |  | 1 | 1 | 2 | 3 | 1 |  | 6 | 16 |  | 41 | 2 | **82** |
| 6 | Wall  Paper | 1 | 1 |  |  |  |  | 1 |  |  |  |  |  |  | 4 |  | **7** |
| 7 | Oasis-Feng/Island | 17 | 2 |  |  |  | 4 |  |  |  |  |  |  |  |  | 3 | **26** |
| 8 | Netflix-5-4-0-Build | 60 | 7 |  | 2 |  |  |  |  |  | 5 | 5 |  |  |  |  | **79** |
| 9 | Remainder | 11 | 4 |  |  |  | 1 | 2 |  |  |  | 4 | 7 | 5 |  |  | **34** |
| 10 | Sound-picker | 9 | 1 |  |  |  |  |  |  |  |  |  |  |  |  | 2 | **12** |
| 11 | Air-Command | 8 | 1 |  |  | 1 |  |  |  |  |  |  |  |  |  |  | **10** |
| 12 | Lifesum-Healthy-Lifestyle | 5 | 1 |  |  |  |  | 1 |  | 4 |  |  | 5 | 1 | 2 | 2 | **21** |
| 13 | Background-Defocus | 10 | 4 |  |  |  | 4 | 1 |  |  |  | 10 |  |  | 6 |  | **35** |
| 14 | Gasbuddy-Find-Cheap-Gas | 11 | 4 |  | 1 |  | 2 |  |  | 1 |  |  | 7 | 2 |  | 3 | **31** |
| 15 | Soundcloud -Music-Audio | 6 | 4 |  |  |  |  |  | 2 |  |  |  |  | 8 | 1 | 2 | **23** |
| 16 | Network-Monitor-Mini | 7 | 2 |  |  |  | 1 | 2 |  |  |  |  | 3 |  |  |  | **15** |
| 17 | Casper Android | 6 | 4 |  |  |  |  |  | 3 |  |  | 20 |  | 6 |  |  | **39** |
| 18 | Line | 15 | 1 |  |  |  | 1 | 1 |  |  |  |  | 6 |  | 2 | 1 | **27** |
| 19 | Diagnoses | 1 |  |  |  |  |  |  |  |  |  |  | 2 | 1 |  |  | **4** |
| 20 | Viber | 42 | 4 |  |  | 1 | 1 |  |  | 1 |  | 9 |  | 7 | 5 |  | **69** |
| 21 | Whats  App | 5 | 1 |  |  |  |  | 2 |  |  |  | 30 |  | 2 |  | 2 | **42** |
| 22 | Firefox | 40 | 4 |  |  |  | 1 | 1 | 4 |  |  |  |  | 8 |  | 1 | **59** |
| 23 | Email And Calendar | 15 | 2 |  |  |  | 1 |  |  |  |  | 108 | 2 |  |  |  | **128** |
| 24 | Google Camera | 9 | 1 |  |  |  |  |  | 1 |  |  | 15 | 8 |  | 1 | 1 | **36** |
| 25 | You  Tube | 21 | 4 |  |  |  | 3 |  |  |  |  | 3 | 3 |  | 3 | 2 | **39** |
| 26 | True Caller | 31 | 2 |  |  |  |  | 2 |  |  |  | 17 | 5 |  | 1 |  | **58** |
| 27 | Samsung Gallery | 12 |  |  |  |  |  |  | 1 |  |  |  | 9 | 3 |  | 1 | **26** |
| 28 | Twitter | 6 | 2 |  |  |  |  | 1 |  |  |  | 15 | 6 | 1 | 1 |  | **32** |
| 29 | Chrome Browser | 1 | 4 | 1 |  |  |  |  |  |  |  | 9 | 5 |  | 12 |  | **32** |
| **#of appearance** | | **522** | **81** | **5** | **5** | **5** | **81** | **20** | **16** | **9** | **5** | **270** | **92** | **45** | **81** | **25** | **1262** |

The detected anti-patterns in each app

| Class#  ant. | # Associations ant. | # Operations ant. | #Namespace ant. | #Attributes ant. | Mobile apps |
| --- | --- | --- | --- | --- | --- |
| 9 | 18 | 0 | 3 | 0 | Test  DPC 4.0.5 |
| 207 | 14 | 0 | 17 | 2 | Avast Android Mobile Security |
| 5 | 8 | 0 | 3 | 0 | Free-Calls-Messages |
| 5 | 1 | 0 | 2 | 0 | Beautiful Gallery 2.1 |
| 9 | 71 | 0 | 2 | 1 | Play Store |
| 1 | 5 | 0 | 1 | 0 | Wall Paper |
| 21 | 3 | 0 | 2 | 0 | Oasis-Feng/Island |
| 60 | 5 | 5 | 9 | 0 | Netflix-5-4-0-Build |
| 12 | 18 | 0 | 4 | 0 | Remainder |
| 9 | 2 | 0 | 1 | 0 | Sound-picker |
| 8 | 1 | 0 | 1 | 0 | Air-Command |
| 5 | 11 | 0 | 1 | 4 | Lifesum-Healthy-Lifestyle |
| 14 | 17 | 0 | 4 | 0 | Background-Defocus |
| 13 | 12 | 0 | 5 | 1 | Gasbuddy-Find-Cheap-Gas |
| 6 | 13 | 0 | 4 | 0 | Soundcloud -Music-Audio |
| 8 | 5 | 0 | 2 | 0 | Network-Monitor-Mini |
| 6 | 29 | 0 | 4 | 0 | Casper Android |
| 16 | 10 | 0 | 1 | 0 | Line |
| 1 | 3 | 0 | 0 | 0 | Diagnoses |
| 43 | 22 | 0 | 4 | 1 | Viber |
| 5 | 36 | 0 | 1 | 0 | Whats  App |
| 41 | 14 | 0 | 4 | 0 | Firefox |
| 16 | 110 | 0 | 2 | 0 | Email And Calendar |
| 9 | 26 | 0 | 1 | 0 | Google Camera |
| 24 | 11 | 0 | 4 | 0 | You  Tube |
| 31 | 25 | 0 | 2 | 0 | True Caller |
| 12 | 14 | 0 | 0 | 0 | Samsung Gallery |
| 6 | 24 | 0 | 2 | 0 | Twitter |
| 1 | 26 | 0 | 5 | 0 | Chrome Browser |
| 603 | 554 | 5 | 91 | 9 | Total |

The detected anti-patterns of each app according to anti-patterns groups

| Protege | OLED | Modelio | Mobile apps |
| --- | --- | --- | --- |
| 17 | 17 | 13 | Test  DPC 4.0.5 |
| 225 | 11 | 229 | Avast Android Mobile Security |
| 9 | 5 | 11 | Free-Calls-Messages |
| 7 | 0 | 8 | Beautiful Gallery 2.1 |
| 28 | 64 | 18 | Play  Store |
| 3 | 4 | 3 | Wall  Paper |
| 23 | 3 | 23 | Oasis-Feng/Island |
| 67 | 5 | 74 | Netflix-5-4-0-Build |
| 25 | 16 | 18 | Remainder |
| 10 | 2 | 10 | Sound-picker |
| 9 | 0 | 10 | Air-Command |
| 12 | 10 | 11 | Lifesum-Healthy-Lifestyle |
| 19 | 16 | 19 | Background-Defocus |
| 24 | 12 | 18 | Gasbuddy-Find-Cheap-Gas |
| 10 | 11 | 12 | Soundcloud -Music-Audio |
| 15 | 3 | 12 | Network-Monitor-Mini |
| 10 | 26 | 13 | Casper Android |
| 24 | 9 | 18 | Line |
| 3 | 3 | 1 | Diagnoses |
| 47 | 21 | 49 | Viber |
| 8 | 34 | 8 | Whats  App |
| 46 | 9 | 50 | Firefox |
| 20 | 110 | 18 | Email And Calendar |
| 18 | 25 | 11 | Google Camera |
| 31 | 11 | 28 | You  Tube |
| 40 | 23 | 35 | True Caller |
| 21 | 13 | 13 | Samsung Gallery |
| 15 | 23 | 9 | Twitter |
| 10 | 26 | 6 | Chrome Browser |
| 763 | 512 | 749 | Total |

The detected anti-pattern in each app according to the detection tool

| Protege | OLED | Modelio | Anti. groups | Mobile apps |
| --- | --- | --- | --- | --- |
| 0 | 0 | 0 | 1 | 1 |
| 2 | 0 | 3 | 2 | 1 |
| 0 | 0 | 0 | 3 | 1 |
| 6 | 17 | 1 | 4 | 1 |
| 9 | 0 | 9 | 5 | 1 |
| 17 | 17 | 13 |  | Total |
| 0 | 0 | 2 | 1 | 2 |
| 15 | 0 | 17 | 2 | 2 |
| 0 | 0 | 0 | 3 | 2 |
| 3 | 11 | 3 | 4 | 2 |
| 207 | 0 | 207 | 5 | 2 |
| 225 | 11 | 229 |  | Total |
| 0 | 0 | 0 | 1 | 3 |
| 1 | 0 | 3 | 2 | 3 |
| 0 | 0 | 0 | 3 | 3 |
| 3 | 5 | 3 | 4 | 3 |
| 5 | 0 | 5 | 5 | 3 |
| 9 | 5 | 11 |  | Total |
| 0 | 0 | 0 | 1 | 4 |
| 2 | 0 | 2 | 2 | 4 |
| 0 | 0 | 0 | 3 | 4 |
| 0 | 0 | 1 | 4 | 4 |
| 5 | 0 | 5 | 5 | 4 |
| 7 | 0 | 8 |  | Total |
| 0 | 0 | 1 | 1 | 5 |
| 1 | 0 | 2 | 2 | 5 |
| 0 | 0 | 0 | 3 | 5 |
| 18 | 64 | 6 | 4 | 5 |
| 9 | 0 | 9 | 5 | 5 |
| 28 | 64 | 18 |  | Total |
| 0 | 0 | 0 | 1 | 6 |
| 1 | 0 | 1 | 2 | 6 |
| 0 | 0 | 0 | 3 | 6 |
| 1 | 4 | 1 | 4 | 6 |
| 1 | 0 | 1 | 5 | 6 |
| 3 | 4 | 3 |  | Total |
| 0 | 0 | 4 | 1 | 7 |
| 2 | 0 | 2 | 2 | 7 |
| 0 | 0 | 0 | 3 | 7 |
| 0 | 3 | 0 | 4 | 7 |
| 21 | 0 | 21 | 5 | 7 |
| 23 | 3 | 23 |  | Total |
| 0 | 0 | 0 | 1 | 8 |
| 7 | 0 | 9 | 2 | 8 |
| 0 | 0 | 5 | 3 | 8 |
| 0 | 5 | 0 | 4 | 8 |
| 60 | 0 | 60 | 5 | 8 |
| 67 | 5 | 74 |  | Total |
| 0 | 0 | 1 | 1 | 9 |
| 4 | 0 | 4 | 2 | 9 |
| 0 | 0 | 0 | 3 | 9 |
| 9 | 16 | 2 | 4 | 9 |
| 12 | 0 | 12 | 5 | 9 |
| 25 | 16 | 18 |  | Total |
| 0 | 0 | 0 | 1 | 10 |
| 1 | 0 | 1 | 2 | 10 |
| 0 | 0 | 0 | 3 | 10 |
| 0 | 2 | 0 | 4 | 10 |
| 9 | 0 | 9 | 5 | 10 |
| 10 | 2 | 10 |  | Total |
| 0 | 0 | 0 | 1 | 11 |
| 1 | 0 | 1 | 2 | 11 |
| 0 | 0 | 0 | 3 | 11 |
| 0 | 0 | 1 | 4 | 11 |
| 8 | 0 | 8 | 5 | 11 |
| 9 | 0 | 10 |  | Total |
| 0 | 0 | 4 | 1 | 12 |
| 1 | 0 | 1 | 2 | 12 |
| 0 | 0 | 0 | 3 | 12 |
| 6 | 10 | 1 | 4 | 12 |
| 5 | 0 | 5 | 5 | 12 |
| 12 | 10 | 11 |  | Total |
| 0 | 0 | 0 | 1 | 13 |
| 4 | 0 | 4 | 2 | 13 |
| 0 | 0 | 0 | 3 | 13 |
| 1 | 16 | 1 | 4 | 13 |
| 14 | 0 | 14 | 5 | 13 |
| 19 | 16 | 19 |  | Total |
| 0 | 0 | 1 | 1 | 14 |
| 4 | 0 | 5 | 2 | 14 |
| 0 | 0 | 0 | 3 | 14 |
| 7 | 12 | 0 | 4 | 14 |
| 13 | 0 | 13 | 5 | 14 |
| 24 | 12 | 19 |  | Total |
| 0 | 0 | 0 | 1 | 15 |
| 4 | 0 | 4 | 2 | 15 |
| 0 | 0 | 0 | 3 | 15 |
| 0 | 11 | 2 | 4 | 15 |
| 6 | 0 | 6 | 5 | 15 |
| 10 | 11 | 12 |  | Total |
| 0 | 0 | 0 | 1 | 16 |
| 2 | 0 | 2 | 2 | 16 |
| 0 | 0 | 0 | 3 | 16 |
| 5 | 3 | 2 | 4 | 16 |
| 8 | 0 | 8 | 5 | 16 |
| 15 | 3 | 12 |  | Total |
| 0 | 0 | 0 | 1 | 17 |
| 4 | 0 | 4 | 2 | 17 |
| 0 | 0 | 0 | 3 | 17 |
| 0 | 26 | 3 | 4 | 17 |
| 6 | 0 | 6 | 5 | 17 |
| 10 | 26 | 13 |  | Total |
| 0 | 0 | 0 | 1 | 18 |
| 1 | 0 | 1 | 2 | 18 |
| 0 | 0 | 0 | 3 | 18 |
| 7 | 9 | 1 | 4 | 18 |
| 16 | 0 | 16 | 5 | 18 |
| 24 | 9 | 18 |  | Total |
| 0 | 0 | 0 | 1 | 19 |
| 0 | 0 | 0 | 2 | 19 |
| 0 | 0 | 0 | 3 | 19 |
| 2 | 3 | 0 | 4 | 19 |
| 1 | 0 | 1 | 5 | 19 |
| 3 | 3 | 1 |  | Total |
| 0 | 0 | 1 | 1 | 20 |
| 4 | 0 | 4 | 2 | 20 |
| 0 | 0 | 0 | 3 | 20 |
| 0 | 21 | 1 | 4 | 20 |
| 43 | 0 | 43 | 5 | 20 |
| 47 | 21 | 49 |  | Total |
| 0 | 0 | 0 | 1 | 21 |
| 1 | 0 | 1 | 2 | 21 |
| 0 | 0 | 0 | 3 | 21 |
| 2 | 34 | 2 | 4 | 21 |
| 5 | 0 | 5 | 5 | 21 |
| 8 | 34 | 8 |  | Total |
| 0 | 0 | 0 | 1 | 22 |
| 4 | 0 | 4 | 2 | 22 |
| 0 | 0 | 0 | 3 | 22 |
| 1 | 9 | 4 | 4 | 22 |
| 41 | 0 | 41 | 5 | 22 |
| 46 | 9 | 50 |  | Total |
| 0 | 0 | 0 | 1 | 23 |
| 2 | 0 | 2 | 2 | 23 |
| 0 | 0 | 0 | 3 | 23 |
| 2 | 110 | 0 | 4 | 23 |
| 16 | 0 | 16 | 5 | 23 |
| 20 | 110 | 18 |  | Total |
| 0 | 0 | 0 | 1 | 24 |
| 1 | 0 | 1 | 2 | 24 |
| 0 | 0 | 0 | 3 | 24 |
| 8 | 25 | 1 | 4 | 24 |
| 9 | 0 | 9 | 5 | 24 |
| 18 | 25 | 11 |  | Total |
| 0 | 0 | 0 | 1 | 25 |
| 4 | 0 | 4 | 2 | 25 |
| 0 | 0 | 0 | 3 | 25 |
| 3 | 11 | 0 | 4 | 25 |
| 24 | 0 | 24 | 5 | 25 |
| 31 | 11 | 28 |  | Total |
| 0 | 0 | 0 | 1 | 26 |
| 2 | 0 | 2 | 2 | 26 |
| 0 | 0 | 0 | 3 | 26 |
| 7 | 23 | 2 | 4 | 26 |
| 31 | 0 | 31 | 5 | 26 |
| 40 | 23 | 35 |  | Total |
| 0 | 0 | 0 | 1 | 27 |
| 0 | 0 | 0 | 2 | 27 |
| 0 | 0 | 0 | 3 | 27 |
| 9 | 13 | 1 | 4 | 27 |
| 12 | 0 | 12 | 5 | 27 |
| 21 | 13 | 13 |  | Total |
| 0 | 0 | 0 | 1 | 28 |
| 2 | 0 | 2 | 2 | 28 |
| 0 | 0 | 0 | 3 | 28 |
| 7 | 23 | 1 | 4 | 28 |
| 6 | 0 | 6 | 5 | 28 |
| 15 | 23 | 9 |  | Total |
| 0 | 0 | 0 | 1 | 29 |
| 4 | 0 | 5 | 2 | 29 |
| 0 | 0 | 0 | 3 | 29 |
| 5 | 26 | 0 | 4 | 29 |
| 1 | 0 | 1 | 5 | 29 |
| 10 | 26 | 6 |  | Total |

The numbers from 1 to 29 present the mobile apps. From 1 to 5 in the second column present the anti-patterns groups with order (Attributes anti-patterns, Namespace anti-Patterns, Operation anti-patterns, Association anti-patterns, and Class anti-patterns). This table is for doing the one-way ANOVA test using SPSS.
